# Comparison of inertial records during anticipatory postural adjustments obtained with devices of different masses

**DOI:** 10.7717/peerj.15627

**Published:** 2023-07-12

**Authors:** Anderson Antunes da Costa Moraes, Manuela Brito Duarte, Eduardo Veloso Ferreira, Gizele Cristina da Silva Almeida, André dos Santos Cabral, Anselmo de Athayde Costa e Silva, Daniela Rosa Garcez, Givago Silva Souza, Bianca Callegari

**Affiliations:** 1 Laboratory of Human Motricity Studies, Federal University of Para, Belém, PA, Brazil; 2 Center of Biological Science, State University of Para, Belém, PA, Brazil; 3 Post Graduation Program in Human Movement Sciences, Federal University of Para, Belém, PA, Brazil; 4 University Hospital Bettina Ferro de Souza, Federal University of Para, Belém, PA, Brazil; 5 Nucleous of Tropical Medicine, Federal University of Para, Belém, PA, Brazil; 6 Institute of Biological Science, Federal University of Para, Belém, PA, Brazil

**Keywords:** Wireless triaxial accelerometer, Postural adjustment assessments, Gait initiation, IMU, Step initiation, Anticipatory postural adjustments

## Abstract

**Background:**

Step initiation involves anticipatory postural adjustments (APAs) that can be measured using inertial measurement units (IMUs) such as accelerometers. However, previous research has shown heterogeneity in terms of the population studied, sensors used, and methods employed. Validity against gold standard measurements was only found in some studies, and the weight of the sensors varied from 10 to 110 g. The weight of the device is a crucial factor to consider when assessing APAs, as APAs exhibit significantly lower magnitudes and are characterized by discrete oscillations in acceleration paths.

**Objective:**

This study aims to validate the performance of a commercially available ultra-light sensor weighing only 5.6 g compared to a 168-g smartphone for measuring APAs during step initiation, using a video capture kinematics system as the gold standard. The hypothesis is that APA oscillation measurements obtained with the ultra-light sensor will exhibit greater similarity to those acquired using video capture than those obtained using a smartphone.

**Materials and Methods:**

Twenty subjects were evaluated using a commercial lightweight MetaMotionC accelerometer, a smartphone and a system of cameras—kinematics with a reflective marker on lumbar vertebrae. The subjects initiated 10 trials of gait after a randomized command from the experimenter and APA variables were extracted: APAonset, APAamp, PEAKtime. A repeated measures ANOVA with *post-hoc* test analyzed the effect of device on APA measurements. Bland–Altman plots were used to evaluate agreement between MetaMotionC, smartphone, and kinematics measurements. Pearson’s correlation coefficients were used to assess device correlation. Percentage error was calculated for each inertial sensor against kinematics. A paired Student’s t-test compared th devices percentage error.

**Results:**

The study found no significant difference in temporal variables APAonset and PEAKtime between MetaMotionC, smartphone, and kinematic instruments, but a significant difference for variable APAamp, with MetaMotionC yielding smaller measurements. The MetaMotionC had a near-perfect correlation with kinematic data in APAonset and APAamp, while the smartphone had a very large correlation in APAamp and a near-perfect correlation in APAonset and PEAKtime. Bland–Altman plots showed non-significant bias between smartphone and kinematics for all variables, while there was a significant bias between MetaMotionC and kinematics for APAamp. The percentage of relative error was not significantly different between the smartphone and MetaMotionC.

**Conclusions:**

The temporal analysis can be assessed using ultralight sensors and smartphones, as MetaMotionC and smartphone-based measurements have been found to be valid compared to kinematics. However, caution should be exercised when using ultralight sensors for amplitude measurements, as additional research is necessary to determine their effectiveness in this regard.

## Introduction

Step initiation begins with heel off of the initial swing leg and it is preceded by physiological events, commonly termed anticipatory postural adjustments (APAs). During APAs, the center of pressure (COP) initially moves to the initial swing side (medio-lateral displacement) and backward, what contributes to the shift of the center of mass (COM) to the initial stance leg (Bonora et al., 2017; Lee et al., 2018). The process, inherent to gait initiation, can be evaluated and inertial measurement units (IMU), containing accelerometers, can indirectly measure the subject’s body sway through uni- or multi-variate signals during gait (Sprager & Juric, 2015). In this regard, sensors with different characteristics have been tested considering its validity and or reliability when compared with some gold standard of measurement (*i.e*., force platforms or video capture systems) (Martinez-Mendez, Sekine & Tamura, 2011; Millor et al., 2013). When analyzing the existing previous literature (see Supplemental Table), a heterogeneity of population, number and type of sensors, and different employing methods is observed. Among the observed limitations, the validity against gold standard measurements was identified only in some studies (Mancini et al., 2016; da Costa Moraes et al., 2022) and the sensors weight vary from 10 to 110 g and sometimes are not reported. The weight of an accelerometer is defined as its mass or the amount of matter it contains. While there is no literature specifically evaluating the weight of sensors for gait analysis, previous studies that examined hand tremors have reported that adding mass loading affects the spectral distribution of the tremor power (Raethjen et al., 2000; Santos et al., 2022). Therefore, the weight of the device in which the accelerometer is embedded is a crucial factor to consider for ensuring its validity, particularly when used to assess anticipatory postural adjustments (APAs). In contrast to focal movements, APAs exhibit significantly lower magnitudes and are characterized by discrete oscillations in acceleration paths. Recently, most sensors have been manufactured with an extremely lightweight profile, while being integrated into smartphones, which may weigh several hundred grams. This development raises concerns about whether differences in the weight of sensors could contribute to an increase in the signal-to-noise ratio, thus compromising the validity of the devices.

The objective of this study is to assess and compare the performance of a commercially available ultra-light sensor, weighing only 5.6 g, with a 168-g smartphone, for measuring APAs during step initiation. Both devices will be compared to a gold standard dataset obtained using a video capture kinematics system. The tendency of an object to resist changes in its state of motion varies with its mass; a more massive object has greater inertia and, thus, a greater tendency to resist changes in motion. As APAs oscillations have minimal energy, it is expected that an ultra-light sensor would be more responsive to these movements compared to a sensor embedded in a smartphone. Therefore, it is hypothesized that APA oscillation measurements obtained with the ultra-light sensor would exhibit greater similarity to those acquired using video capture than those obtained using a smartphone.

## Methods

Data were collected as previously described in our previous study (da Costa Moraes et al., 2022). Specifically, we use the same equipment, procedures for conducting the experiment, and for recording, processing, and marking the events from the beginning of the step.

### Subjects

The participants of this study included twenty healthy individuals of both sexes (11 F; 9 M), aged 18 to 40 years, with participants having a mean age of 29.6 ± 6.7 years, mean height of 1.7 ± 0.08 m, and mean weight of 72.8 ± 14.3 kg. Participants agreed to participate in the research and signed a written informed consent. The Ethics Committee approved all procedures used in this research of the Federal University of Para, Institute of Health Sciences under opinion 3.773.655 CAEE: 25667919.2.0000.0018. The participants were recruited by convenience. Research screening and evaluations were conducted in the Human Movement Laboratory (LEMOH) of the Federal University of Para, located at Avenida Generalíssimo Deodoro, #1 Zip Code 66055-240, Belem-PA.

Participants were right-footedness. Individuals with neurological problems, vestibular disorders, altered gait, and orthopedic trauma were excluded from the study. Subjects with corrected vision wore glasses during the experiment (Martinez-Mendez, Sekine & Tamura, 2011).

### Instruments

Three instruments (Smartphone, kinematics, and accelerometer) were simultaneously used to measure and record the COM accelerations during gait initiation. We proceeded a video capture of a reflective marker that was fixed on the fifth vertebra of the lumbar spine (L5) at a sampling frequency of 120 Hz to record the COM accelerations in the analysis axis of a three-dimensional system with three cameras (Simi; Simi Motion, Unterschleißheim, Germany). A wireless inertial sensor (MetaMotionC; Mbientlab, San Francisco, CA, USA) and a smartphone (Android A10s; Samsung, Seoul, Korea) positioned at L5 was also used. MetaMotionC is a triaxial device, approximately 25 mm × 4 mm in diameter, ultralight with only 5.6 g, replaceable 200 mAH battery, with data transfer *via* Bluetooth Low Energy Smart®. The smartphone has a weight of 168 g and is equipped with an integrated tri axial accelerometer and 2- and 1.5-GHz speed Octa Core processors. An app called “*Momentum Science*” (*Momentum Science app*, Belem, Brazil) was used for registration to assess the accelerations of body oscillations. The acceleration data in the X, Y, and Z axis were collected at 100 Hz. A sensor, an on-off Foot Switch (FS), with an acquisition rate of 2,000 Hz (EMG System do Brasil, Ltda., São José dos Campos, SP, Brazil), was used to mark the heel off (Jasiewicz et al., 2006).

### Experimental protocol

**Figure 1:**
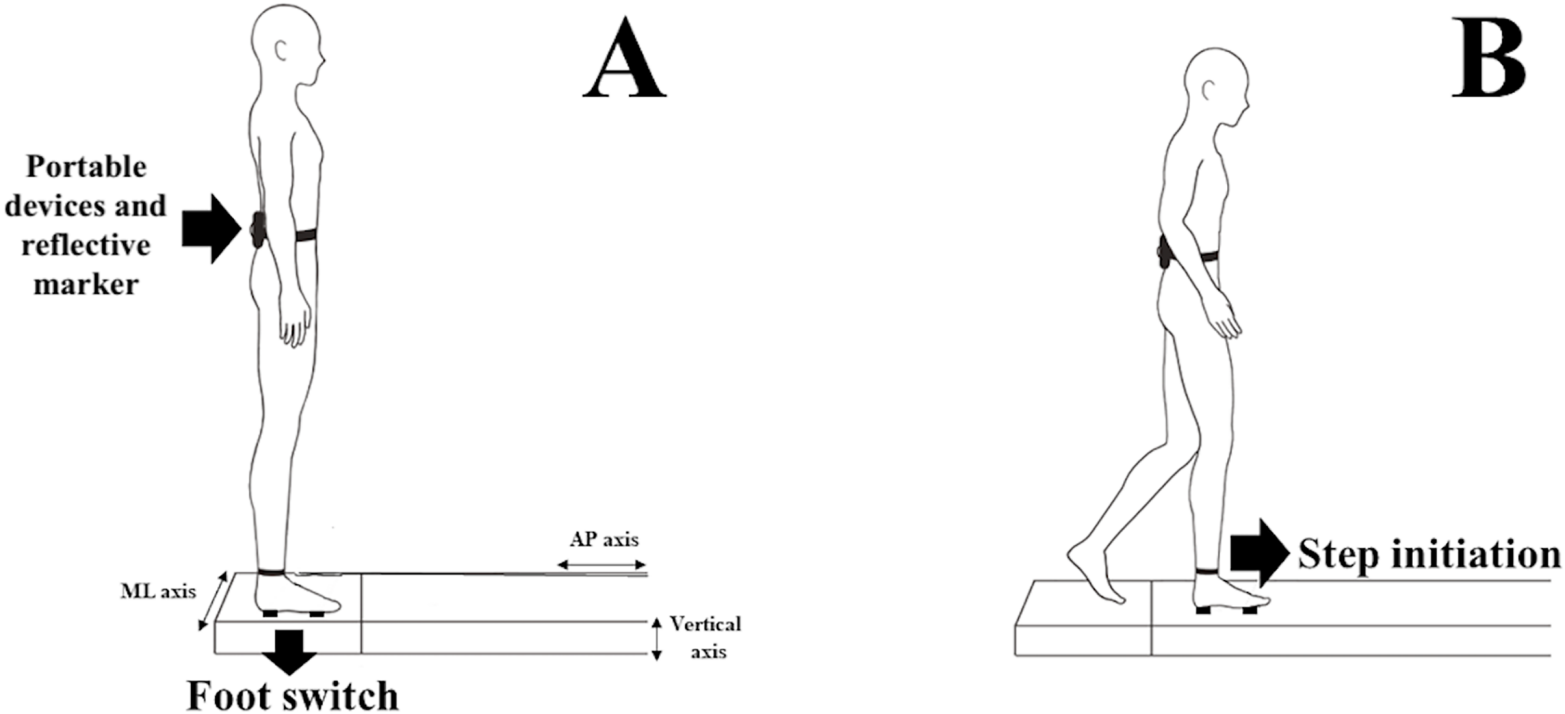
(A) Subject standing bipedally on the platform with the devices at L5 vertebra and Footswitch (FS) at the base of the calcaneus and head of the second metatarsal. (B) Subject initiating the step with the right leg towards the 2-m walkway after the researcher’s command.

The subjects were instructed to stand barefoot in a bipedal stance with their arms at their sides. The devices were fixed to the region of the fifth vertebra of the lumbar spine (L5) with a neoprene strap. A reflective marker was positioned over it to acquire the kinematic system footage (Fig. 1). The FS was fixed on each individual’s right forefoot and hindfoot to mark the moment when the heel leaves the ground and the support phase of the step.

**Figure 2:**
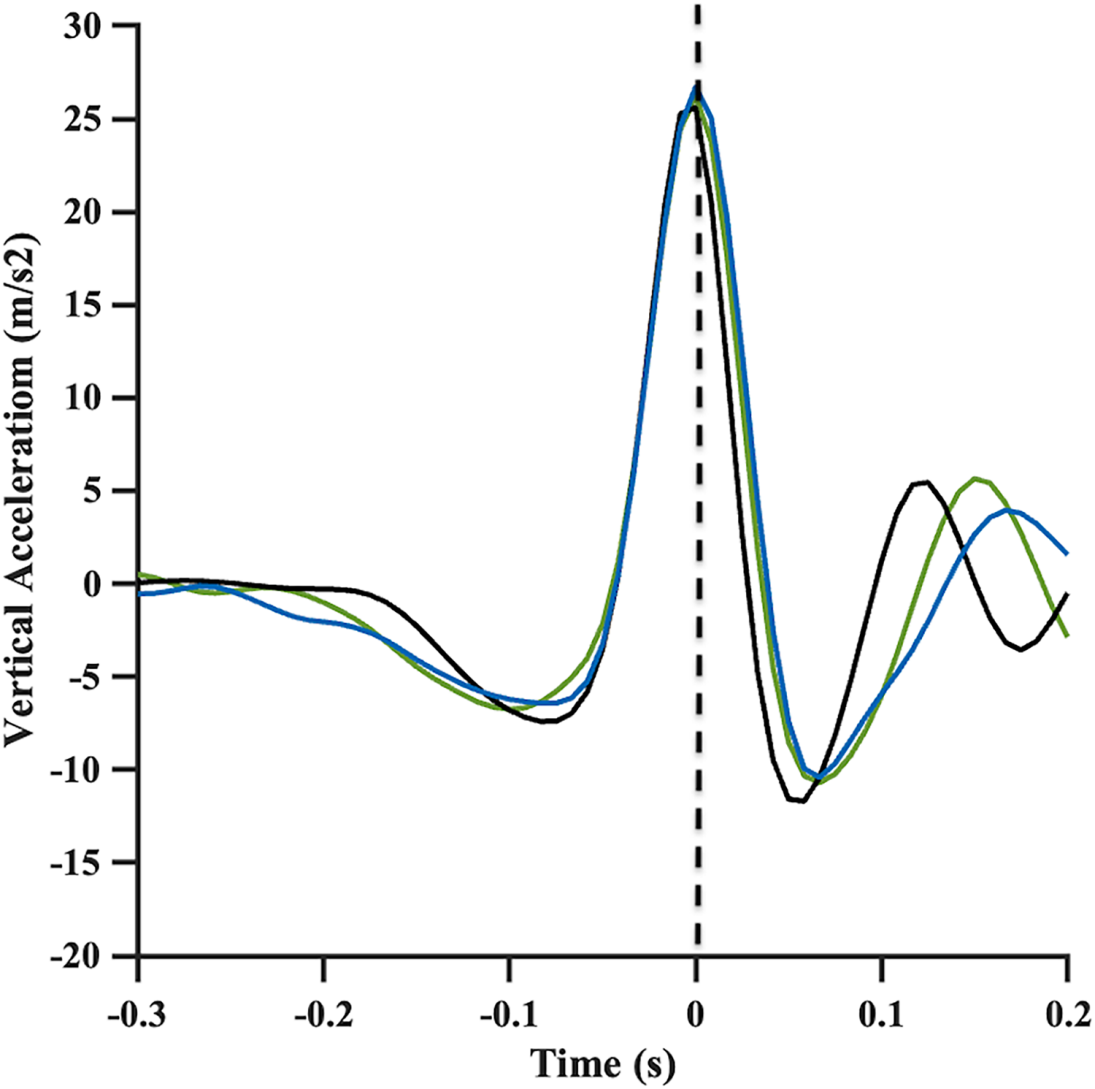
Vertical acceleration signals recorded bythe smartphone (black line), *MetaMotionC* (green line) and by kinematics (blue line) during vertical jump. Dotted line represents peak acceleration on this axis, corresponding to impact with ground, which is used for synchronization. Acc, Acceleration.

Initially, the subjects were instructed to jump vertically in the same place. The records were then aligned by the peak of the signal in the vertical axis, which characterizes the moment of impact with the ground to synchronize the two evaluation instruments (Fig. 2).

Subsequently, the subjects stood on the 2-m walkway before starting the experiment. The feet were placed on marks drawn to control the foot position during gait. The heels were separated mid-laterally by 6 cm; measured with a tape measure. The subject focused on a mark at eye level on the wall at a distance of 3 m and stepped forward when he/she heard a command from the researcher. The time of the command was self-selected without prior announcement. Before the recording, to help gain familiarity, the subjects were asked to perform a step. Ten trials were performed, starting with the right lower limb, where the FS was fixed. The protocol was performed twice, on alternate days, with 1 week between them. The mean value between these measurements was used for further analysis.

### Signal processing

**Figure 3:**
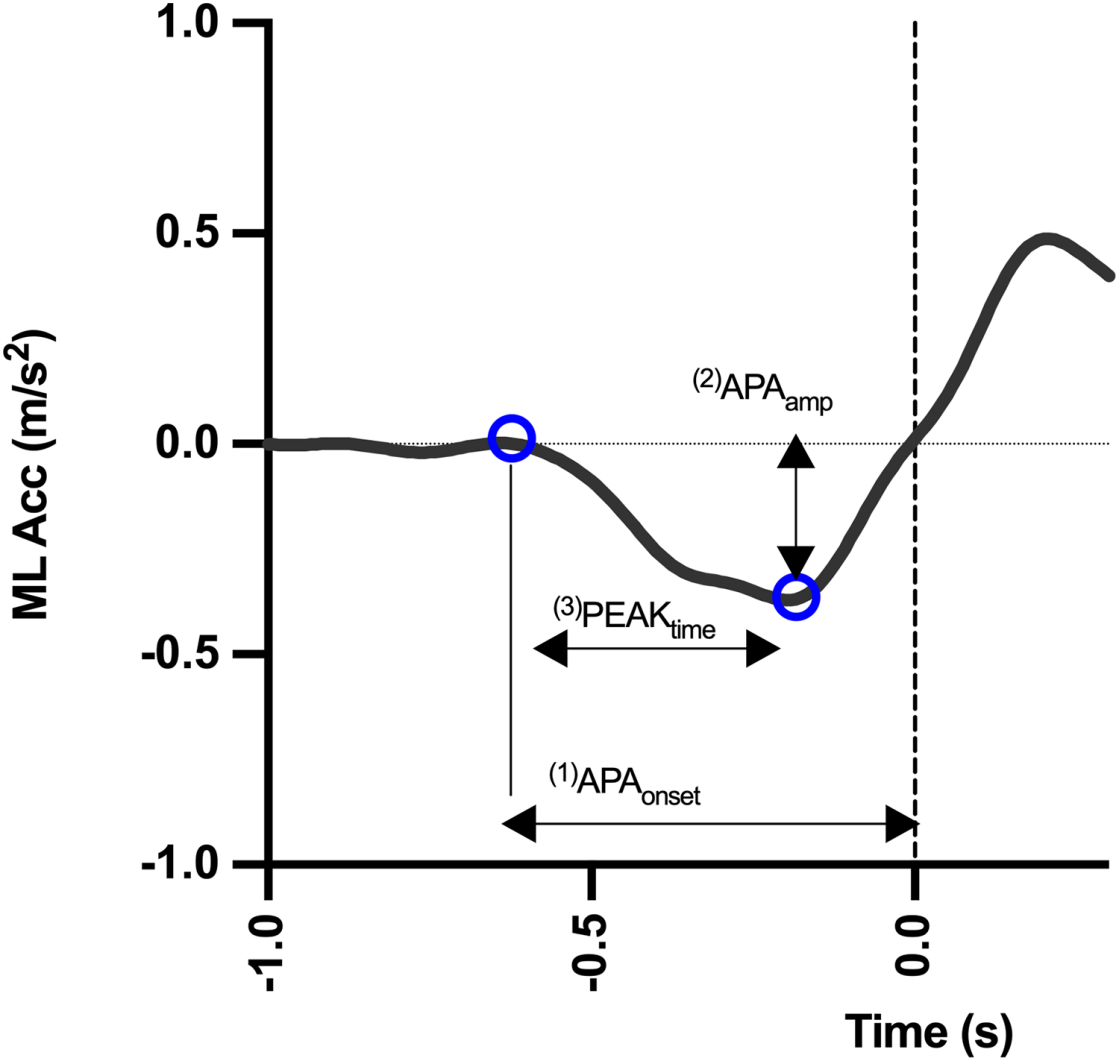
Mid-lateral acceleration curve and measurement variables. The dotted line represents the moment when the heel leaves the ground.

The data acquired by the equipment were synchronized for comparison. The synchronization and offline analysis were done using the MATLAB program (MathWorks, Natick, MA, USA) from the subject’s initial jump. The FS set the moment of heel off. Subsequently, the trials within each series were calculated for each individual. The COM accelerations in the mid-lateral direction were analyzed, extracted from the recordings of the measuring instruments. The raw data coordinates in the lateral mid-axis were generated from video analysis, smartphone and MetaMotionC. A 30-Hz second-order low pass Butterworth was used. The step parameters were calculated and exported (Fig. 3):

(1) APA_onset_: is the APA latency, the moment when the first mid-lateral deviation occurs in acceleration data that exceeds two standard deviations above the baseline.
(2) APA_amp_: comprises the maximum mid-lateral acceleration of the COM before the exit of the heel.
(3) PEAK_time_: the time it takes to reach maximum acceleration amplitude.

### Statistical analysis

Statistical analysis was conducted with GraphPad PRISM 9 software. A pilot study performed in the first six subjects was performed and the mean and standard deviation for the APA_onset_ (s) was calculates. The mean difference between sessions was achieved as 0.085 ± 0.099 s. The sample size was calculated using 80% statistical power and 95% confidence interval. A minimum required sample size of 13 individuals was calculated. The Shapiro–Wilk test was conducted to analyze the normality of the variables. The data were described in a table in terms of mean and standard deviation, for each parameter. To compare the absolute values measured with MetaMotionC, smartphone and Kinematic, a repeated measures ANOVA, followed by tukey *post-hoc* test, was used to evaluate the effect of the factor device on APA measurements. Agreement between the measurements done using MetaMotionC and smartphones against measurements done by kinematics was further analyzed by Bland–Altman plots which the horizontal central line (mean) indicates the bias and the outer lines (±1.96 × standard deviation) indicate the limits of agreements (LoA). The 95% confidence interval (CI) on bias was calculated, and bias was considered significant if bias equal to zero was out of in the CI (Jensen et al., 2008).

Finally, a linear person’s correlation was performed between the devices (MetaMotionC or smartphone) and Kinematic. Pearson’s correlation coefficients (r) were interpreted with magnitude thresholds of 0–0.1: trivial; 0.1–0.3: small; 0.3–0.5: moderate; 0.5–0.7: large; 0.7–0.9: very large and 0.9–.0: near perfect.

To compare the measurements of each inertial sensor to the gold standard, the percentage of relative error was calculated between MetaMotionC and kinematics and between the smartphone and kinematics for all variables, following the equation bellow:

$Percentage\; Error = \displaystyle{{\left| {inertial\; sensor - kinematics} \right|} \over {kinematics}} \times 100$

A paired Student’s t-test comparing the MetaMotionC and smartphone’s percentage error was employed. Significance was determined at the level of *p* < 0.05.

## Results

Table 1 displays the mean values for each variable examined in this study. The results indicate that there was no statistically significant difference between the three instruments for the temporal variables APA_onset_ and PEAK_time_. In contrast, a significant difference was observed for the variable APA_amp_, with the MetaMotionC device yielding smaller measurements (F_(1.495, 29.89)_ = 7.434; *p* = 0.0049). Further *post hoc* comparisons using the Tukey HSD test revealed that the mean score for the MetaMotionC was significantly lower than those for the smartphone (*p* = 0.002) and the kinematic (*p* = 0.017). However, no significant difference was observed between the smartphone and the kinematic data.

**Table 1:** Analysis of the mean and standard deviation of the subjects in the three instruments: smartphone, kinematics and MetaMotionC.

Variable	MetaMotionC	Kinematics	Smartphone	F	*p*-value
APA_onset_ (s)	−0.617 ± 0.066	−0.620 ± 0.067	−0.619 ± 0.068	1.403	0.258
APA_amp_ (m/s^2^)	−0.249 ± 0.097	−0.275 ± 0.083	−0.295 ± 0.107	7.434	0.004*
PEAK_time_ (s)	0.272 ± 0.072	0.276 ± 0.059	0.290 ± 0.073	1.356	0.269

**Note:**

* *p* < 0.05.

Table 2 displays the Pearson’s correlation coefficients between each device and the kinematic data. The results show that the MetaMotionC device exhibited a near-perfect correlation with the kinematic data in APA_onset_ and APA_amp_, and a large correlation in PEAK_time_. In contrast, the smartphone demonstrated a near-perfect correlation with the kinematic data in APA_onset_, a very large correlation in APA_amp_, and a very large correlation in PEAK_time_.

**Table 2:** Linear correlation between the inertial sensors (MetaMotionC and smartphone) and the kinematics.

Variable	MetaMotionC	Smartphone
APA_onset_ (s)	0.99 (*p* < 0.00001)	0.99 (*p* < 0.00001)
APA_amp_ (m/s^2^)	0.92 (*p* < 0.00001)	0.78 (*p* = 0.0003)
PEAK_time_ (s)	0.84 (*p* < 0.00001)	0.71 (*p* = 0.0003)

**Figure 4:**
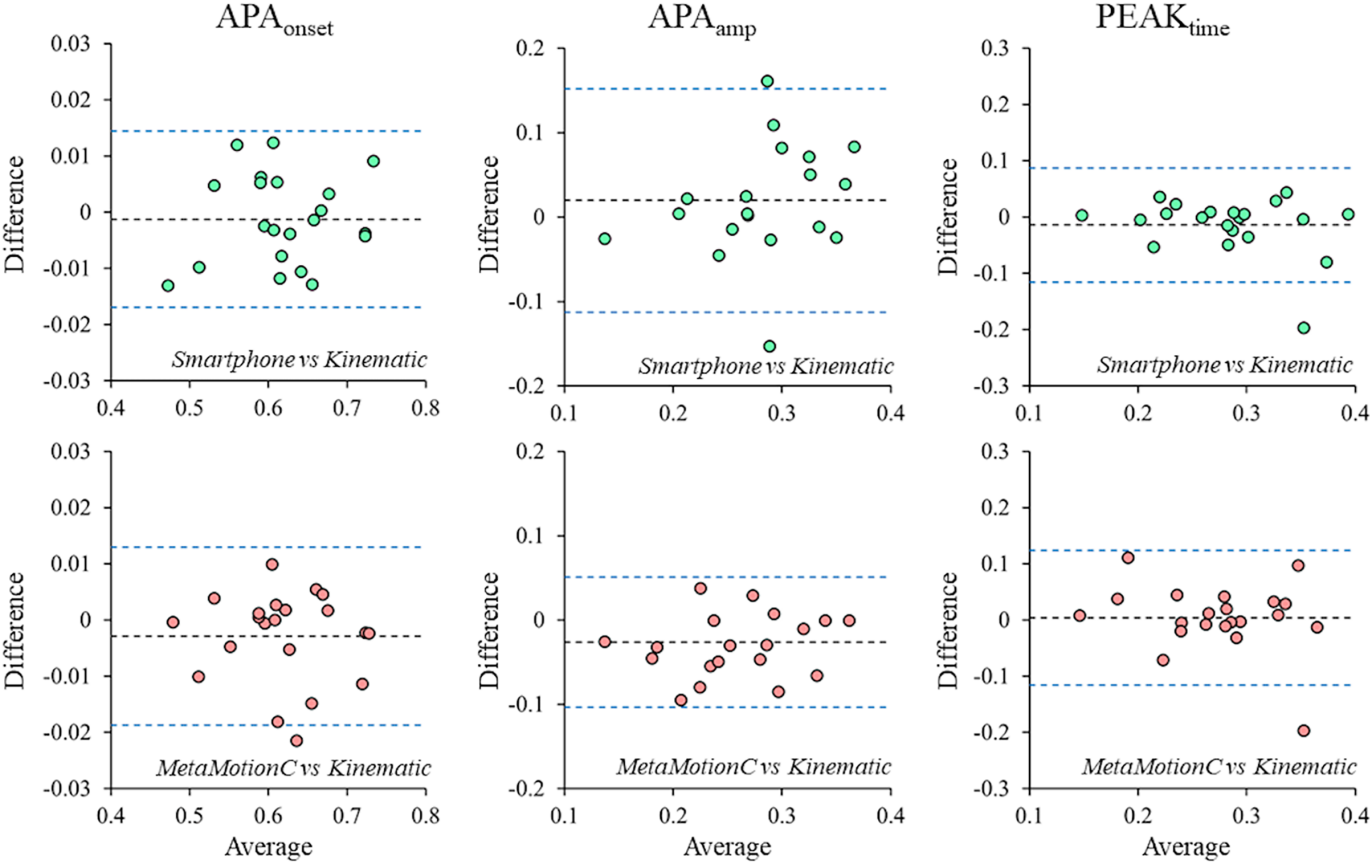
Bland–Altman graphs evidencing the agreement levels between the instruments.

Bland–Altman plots of APA_onset_, APA_amp_ and PEAK_time_ values measured by smartphone and MetaMotionC *vs*. the measurements obtained using kinematics are shown in Fig. 4. For APA_onset_, there were non-significant bias between smartphone and kinematics (mean difference: −0.00122 s; 95% CI on difference: from [0.0022 to −0.0047]; LoA: from 0.0145 to −0.0169), and between MetaMotionC and kinematics (mean difference: −0.00122 s; 95% CI on difference: from [0.00063 to −0.0063]; LoA: from 0.013 to −0.0189). For APA_amp_, it was observed a non-significant bias between smartphone and kinematics (mean difference: −0.019 m/s^2^; 95% CI on difference: from [0.049 to −0.0092]; LoA: from 0.152 to −0.113), while there was a significant bias between MetaMotionC and kinematics (mean difference: −0.026 m/s^2^; 95% CI on difference: from [−0.00934 to −0.043]; LoA: from 0.0051 to −0.103 m/s^2^). For PEAK_time_, there were non-significant bias between smartphone and kinematics (mean difference: −0.0141 s; 95% CI on difference: from [00794 to −0.0361]; LoA: from 0.087 to −0.115), and between MetaMotionC and kinematics (mean difference: 0.0038 s; 95% CI on difference: from [−0.029 to −0.022]; LoA: from 0.124 to −0.116 s).

**Figure 5:**
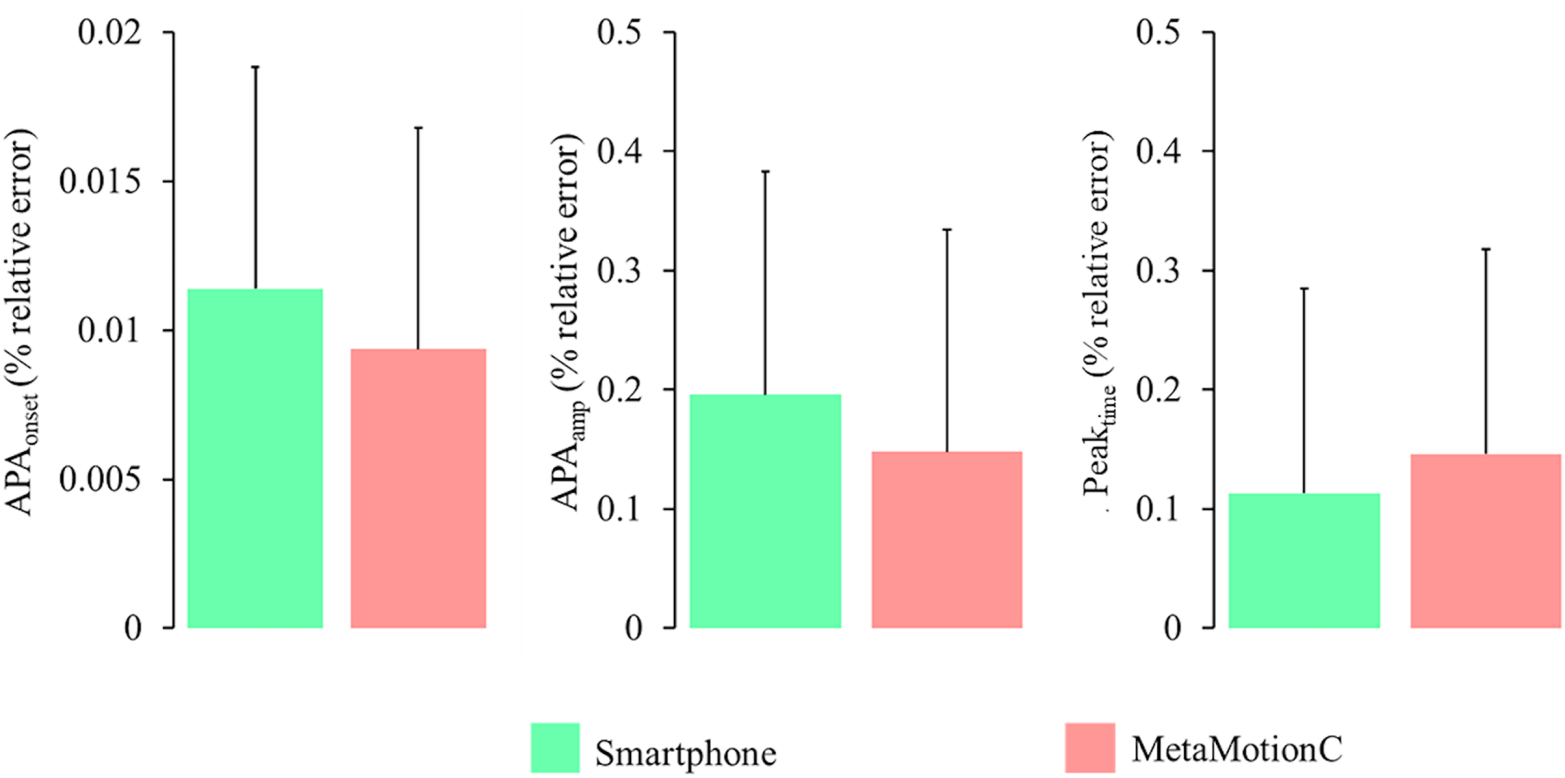
Percentage of relative error calculated between MetaMotionC and kinematics and between the smartphone and kinematics for all variables.

No significant differences in the percentage of relative error were observed between the smartphone and MetaMotionC, as illustrated in Fig. 5.

## Discussion

The purpose of this study was to evaluate and validate the functionality of a commercially available ultra-light sensor, which has a weight of only 5.6 g, when compared to a 168-g smartphone, for measuring APAs during step initiation. Both devices were be compared with a gold standard dataset collected using a video capture kinematics system and we hypothesized that APA oscillation measurements obtained using the ultra-light sensor would demonstrate a greater similarity to those acquired through video capture compared to measurements acquired using a smartphone.

Our main results showed that for the temporal variables, both devices had performance validated with the gold standard, with no difference between the measurements. However, the acceleration amplitude (APA_amp_) measured with the lightweight sensor MetaMotionC was lower compared to the kinematics and smartphone, which led us to reject our initial hypothesis. Despite the correlations with kinematics being large to near perfect and there being no difference in the percentage error measurements between the MetaMotionC or smartphone and the kinematics, the sensor consistently showed lower measurements than the kinematics for the APA_amp_, as confirmed in the Bland-Altman plots.

The several studies that investigated validation and reliability of accelerometers for measuring APA before gait initiation had similarities and important differences that can be debated with our findings. Previously, all studies had used force platforms or walkways with pressure sensors as gold-standard method to validate the inertial sensors for APA before step initiation (Martinez-Mendez, Sekine & Tamura, 2011; Mancini et al., 2016; Gazit et al., 2020; da Costa Moraes et al., 2022). Our approach used video capture to compare to the inertial readings. The advantage of video capture as gold-standard method is that we can compare the accelerometric measurements with acceleration readings of the body movements before the step initiation. Other similarity among the different studies that used accelerometric readings before step initiation was the use of sensors in the lumbar region (Mancini et al., 2009), although it is common the placement of additional sensors in lower limb (Bonora et al., 2015) or other parts of the trunk (Martinez-Mendez, Sekine & Tamura, 2011). All studies found good-to-excellent validation of the accelerometers to measure APA before gait initiation, similar to our study. These results strongly indicate that the mass of the sensor has no significant influence on the validation quality with the gold standard. Our study aligns with these findings, as both devices showed a large to near perfect correlation with the kinematic. However, our study is the first to compare devices with different masses, and our results raise the question that, despite having a good correlation with a gold standard, an ultralight sensor may systematically measure values below, especially in amplitude variables, and its use requires caution.

Some hypotheses as to why a lighter sensor may measure lower values of acceleration amplitude compared to a heavier sensor can be proposed. One possibility is that the lighter sensor may be more susceptible to external factors (noise) such as air resistance or vibrations. For instance, during APAs to start gait analysis, the resistance encountered by the sensor could cause it to move slightly off course or record smaller movements than a heavier sensor, resulting in more interferences and lower measurements of acceleration amplitude. This effect may be particularly relevant in the lumbar region where our sensor was placed, where movements are relatively small compared to other parts of the body. Another possible factor to consider when measuring lumbar acceleration during gait is the signal-to-noise ratio of the sensor. The signal-to-noise ratio refers to the proportion of the useful signal (in this case, the acceleration amplitude being measured) to the unwanted noise in the measurement signal. A lower signal-to-noise ratio can result in less accurate measurements of acceleration amplitude, particularly if the sensor is lightweight and more sensitive to external factors that can introduce noise into the measurement.

Finally, in the context of measuring lumbar acceleration during gait, it’s important to consider how the mass of the sensor could affect the measurement results. One potential concern is that the differences in mass between sensors could affect the way they interact with the lumbar region during movement. A heavier sensor may apply more force to the lumbar area, resulting in higher measurements of acceleration amplitude. Conversely, a lighter sensor may not apply enough force to accurately capture the amplitude of movement. Thus, the mass of the sensor should be taken into account when analyzing the measurement data, especially if comparing results from sensors with different masses.

This emphasizes the importance of considering the weight of an accelerometer when selecting a sensor for gait analysis. Differences in weight can have an impact on the device’s performance and accuracy, and may lead to an increase in signal noise, thereby compromising its validity and reliability. Therefore, it is crucial to carefully evaluate the weight of accelerometers before selecting one for a specific application.

This research has some limitations, and an important issue to be considered is the imposition of the right foot to start the step what may not allow a natural movement (Dessery et al., 2011). It is already known that the non-preferential limb provides a greater lateral impulse on the ground during gait and we tried to minimize this issue by recruiting right-footedness participants. In addition, we did not control some gait initiation variables such as velocity, cadence, and stride variability, and the lack of an international protocol for gait initiation may have influenced the full acceptance of this study’s hypothesis.

Therefore, we propose that, in future research, new experimental protocols be considered with the analysis of more spatio-temporal gait variables. A global parameterization for the evaluation of gait initiation can be achieved.

## Conclusion

Based on the findings of this study, it can be concluded that both ultralight sensors and smartphones can be utilized to evaluate the temporal analysis in gait initiation, as the validity of MetaMotionC and smartphone-based measurements was observed when compared to kinematics. However, it is important to exercise caution when using ultralight sensors for amplitude measurements, as further research is needed to determine their effectiveness in this area.

## Supplemental Information

10.7717/peerj.15627/supp-1 Supplemental Information 1 Dataset.

10.7717/peerj.15627/supp-2 Supplemental Information 2 Supplementary Table.

## Additional Information and Declarations

### Data Availability

The following information was supplied regarding data availability: The raw data is available in the Supplemental File.
