# Supplementary material for: Comparison of inertial records during anticipatory postural adjustments obtained with devices of different masses
Source: PeerJ. 2023 Jul 12;11:e15627. doi: 10.7717/peerj.15627 (PMC10349560; doi:10.7717/peerj.15627)
Supplement: Supplemental Information 2 [file peerj-11-15627-s002.docx]

Supplementary Table. References of investigations that used accelerometers to record APA before step initiation.

| **Author, year** | **Sensor** | **Manufactor** | **Weight** | **Valitidy** | **Reliability** | **Population** |
| --- | --- | --- | --- | --- | --- | --- |
| Bonora et al, 2015 | Acc and Gyro  Linear acceleration and angular velocity data were sampled at 50 Hz | 2 inertial sensors (TMA, Tecnobody, Dalmine, Italy) | Not specified | Yes/ Force Platform | No | 11 PD and 20 healthy subjects |
| Martinez-Mendez et al, 2011 | Acc and Gyro  Linear acceleration and angular velocity data were sampled at 28 Hz | 2 inertial sensors from the own lab | 110 g | Yes/ Force Platform | Yes  intra test  (5 trials) | 10 subjects (7 men, 3 women) with no previous history of neurological disorders |
| Gazit et al, 2020 | Only Acc sampled at 128 Hz | The triaxial acceleration sensor (Opal by APDM) | <26g | Yes/ Mat | No | 41 healthy young adults and 26 older female adults |
| Mancini et al, 2016 | Experiment I: Only Acc sampled at 50 Hz  Exmeriment II:  Only Acc sampled at 50 Hz | Experiment I  : 3 IMUs (Opals by APDM Inc.)  Experiment II:  3 IMUs (MTX Xsens0 | Experiment I: <26g  Experiment II:  11.2g | Experiment I: Yes/ Kinematics  Experiment II:  No | Experiment I: No  Experiment II:Yes inter test (30 min rest) | Experiment I: 10 persons with mild-to-moderate idiopathic PD e 12 healthy  Experiment II:  17 PD and 17 healthy subjects |
| Moraes et al, 2022 | Acc sampled at 50Hz | Mobile, Android A10s, Samsung. | 168g | Yes/ Kinematics | Yes inter test (one week rest) | 20 healthy individuals |
| Lencioni et al, 2022 | Acc sampled at 140Hz | WaveTrack Inertial System  Cometa, Italy | 10 g | Yes/ Force Platform | No | 25 PD and 8 healthy older subject |
| Fantozzi et al, 2022 | Acc sampled at 258Hz | WaveTrack Inertial System  Cometa, Italy | 10 g | No | No | 10 healthy individuals |
